# The invite study: incisional hernia prevention: prophylactic mesh from the patient’s perspective

**DOI:** 10.1007/s10029-025-03463-z

**Published:** 2025-09-04

**Authors:** Laurie Smith, Laura Knight, Alun Meggy, Tessa Watts, Jared Torkington, Julie Cornish

**Affiliations:** 1 Department of Colorectal Surgery, University Hospital of Wales, Cardiff, UK; 2 Cardiff University, Cardiff, UK; 3 CEDAR Health technology research group, Cardiff, UK

**Keywords:** Prevention, Mesh prophylaxis, Implementation, Incisional Hernia

## Abstract

**Purpose:**

Mesh-augmented abdominal wall closure (Mesh prophylaxis) reduces incisional hernia rates in high-risk patients. In spite of a large body of evidence supporting its efficacy and safety, use of mesh prophylaxis is low in the US and UK, possibly due to negative perceptions of surgical mesh^(1-2)^. This study aimed to assess the acceptability of mesh to patients and determine factors that influence acceptability.

**Methods:**

Following ethical approval, a convergent mixed-methods study was conducted whereby patients who had undergone elective or emergency surgery (*n=332) *were approached to participate in a questionnaire assessing knowledge and opinions regarding mesh prophylaxis Semi-structured interviews were conducted in a subset of participants (*n*=12) and thematic analysis performed.

**Results:**

120 questionnaires were returned with a response rate of 36.1%. The majority (61.8%) of participants had heard of surgical mesh, with half (51.7%) having a negative association, driven by the media. Half (50%) of participants had pre-existing concerns about mesh, however the majority (91%) felt mesh prophylaxis to be acceptable, findings which were echoed in the qualitative component. Analysis of interview data identified three themes: "Knowledge of mesh" and "Acceptability of mesh", which triangulated with findings in the survey data and "Shared decision-making", which explored how participants wanted to receive information about mesh. Factors affecting acceptability of mesh included the nature of information given to patients, and the way in which it was delivered.

**Conclusions:**

Despite negative pre-conceptions, mesh prophylaxis is broadly acceptable to patients. The results of this work will be used to develop patient resources to support mesh prophylaxis. Future mixed-methods studies identifying the surgeons’ barriers to use of mesh are needed to allow targeted implementation of prophylactic mesh.

**Supplementary Information:**

The online version contains supplementary material available at 10.1007/s10029-025-03463-z.

## Introduction

Incisional hernia (IH) is the most common complication of abdominal surgery, with an incidence ranging from 12 to 30% in the literature [1, 2]. IH development is associated with a huge financial burden on healthcare systems, but also a psychological impact on patients, and prevention of this commonly occurring iatrogenic condition is of utmost importance [3–5].

Mesh-augmented abdominal wall closure (mesh prophylaxis) has been used as an adjunct to abdominal wall closure and has been shown to reduce the incidence of IH [6]. A systematic review published in 2023 of 12 randomised control trials found a lower incidence of incisional hernia after mesh placement compared with primary suture closure (11.1% vs. 21.3%, RR = 0.32; 95% CI = 0.19 0.55, *p* < 0.001). Mesh prophylaxis also appears to be safe, with comparable levels of post-operative pain and rates of surgical site infection when compared with suture closure, but increased rates of post-operative seroma formation [7].

Mesh prophylaxis may not be necessary for all patients undergoing midline surgery, rather in patients with higher risk of IH development, such as those with obesity, smokers, previous abdominal surgery or undergoing vascular surgery [1]. Identification of these “Higher risk” patient groups pre-operatively is possible using risk predictors such as the “Penn Hernia Calculator”, although further work is needed to refine these models and quantify what constitutes “high risk” [8, 9].

The European and American Hernia Society guidelines published in 2022, recommended mesh prophylaxis as safe to use in “High risk” patient groups, and although further work is needed to identify optimal mesh type and plane of position, focus should shift towards implementation of this technique to reduce the burden of IH on patients and healthcare services [10].

Despite an increasing body of evidence and guidelines, uptake of mesh prophylaxis is slow. Two published surveys of surgeons across Europe, America and the United Kingdom estimate the proportion of surgeons currently using mesh prophylaxis to be between 3 and 15% [11, 12]. Recent scandals regarding mesh-related complications reported in the media may be factoring into this hesitation, with concern over safety of mesh spilling over from uro-gynaecological procedures into hernia surgery [13]. Negative views on mesh predominate on social media, with most of the content being negative, and vaginal mesh-related lawsuits have paid out nearly 8 billion dollars since 2010 [14, 15].

In summary, it would appear that in spite of the evidence, surgeons are hesitant to use mesh prophylactically. Although the reasons are not entirely clear, recent mesh scandals and concern in the public domain over mesh complications may well be factoring into this decision. To date however, there is no research into patient views on mesh, and this study aims to address this knowledge gap in order to target potential barriers to implementation of prophylactic mesh.

## Methods

This was a single-site, cross-sectional study with a convergent mixed-methods study design. A mixed-methods model, was used as the strengths of both qualitative and quantitative research would add a greater depth of understanding to the information gathered and uncover information that may not have been found using one method alone [16].

The primary study objective was to explore patient perceptions of prophylactic mesh. Secondary objectives were understanding patients’ level of awareness of incisional hernia as a risk of surgery, identifying factors that may change or influence the acceptability of prophylactic mesh.

### Study design

In the quantitative component, a survey was used to capture participants’ views on mesh. In the qualitative component, semi structured interviews were used to further explore participants’ views on prophylactic mesh and to identify and understand factors affecting its acceptability. For this study, a convergent mixed-methods approach was selected whereby qualitative and quantitative arms were conducted concurrently and analysed together, with triangulation of perspectives from qualitative and quantitative data allowing a robust answer to the research question. Further detail of the research methods used can be found in the previously published protocol [17].

Participant identification and recruitment.

All patients who had undergone elective or emergency colonic resection within one University Health board in Wales were considered eligible for inclusion if they were:

- Over the age of 18 years old.
- Able and willing to provide valid informed consent.
- Undergone elective or emergency colonic resection > 12 months ago.

Potential participants were excluded if they:

- Were unable or unwilling to give informed consent.
- Had a palliative diagnosis either at time of surgery, or since.
- Were unable to understand or complete study questionnaires due to intellectual or cognitive impairment or due to insufficient English-language skills.

### Participant identification, recruitment and consent

For the quantitative component, eligible patients were identified by a member of the study team through local databases of elective colorectal cancer resections and the national emergency laparotomy audit (NELA) database from a single institution over a three-year period (2017–2020).

Potential participants were sent a copy of the consent form, information leaflet and the survey, along with a pre-paid envelope, and were considered enrolled once they had returned both the envelope and consent form. Non-responders were then called to invite them to participate and sent another study pack if they agreed to take part. If there was no answer or no return of the study pack, then they were deemed non-responders and no further attempts at contact were made. Participants were also approached at routine clinic appointments.

### Quantitative component: Survey design and development

Following a search of the literature, no pre-existing validated questionnaires relating to patients’ views on mesh or relating to health behaviours and patient decision-making were found. Therefore, a survey was developed using principles of study design outlined by Oppenheim [18]. The Health Belief Model (HBM), an internationally recognised framework for understanding health beliefs and identifying areas for change, was used as a framework for survey design [19].

The survey instrument was pilot tested on the first ten participants to assess usability. The final questionnaire can be seen in *Appendix 1*.

### Qualitative component: semi-structured interviews

Semi-structured interviews were conducted with a sub-sample of participants who had indicated on their consent forms that they would be willing to participate in an interview.

All participants who indicated they would like to participate in interviews were eligible to participate. However, to ensure a diverse range of opinions and views, participants were purposively sampled, with those recording views pertinent to the research question in the questionnaire prioritised to be approached.

Interviews were conducted via Microsoft Teams^®^ or Zoom^®^ with two interviews conducted in person as per participant preference. All interviews were conducted by a surgeon with experience in semi-structured interviewing but with no direct experience of mesh prophylaxis to reduce possible bias.

An interview guide based on a series of open questions around the themes of mesh and patients’ decision-making behaviour was developed and administered to all participants. Flexibility within the interview guide was encouraged, allowing the opportunity to explore additional topics that came up during the interview.

Data collection continued until saturation, which was defined as no new themes emerging for two consecutive interviews [20]. This occurred after 12 interviews had been completed. Interviews lasted up to 30 min.

### Ethical approval and Patient and Public involvement

Following study set-up and registration (IRAS 310695, ClinicalTrials.gov: NCT05384600) the study received ethical approval from the Research and Ethics Committee for Wales, (Approval number 22/PR/0678). Local Research and Development acted as study sponsor. Patient and Public Involvement (PPI) representatives were included in all aspects of study design, the development of patient information leaflets and in the design and testing of study questionnaires.

### Quantitative analysis plan

Following enrolment, participants’ electronic health record was accessed, and baseline demographics recorded. The Welsh Index of Multiple Deprivation (WIMD) was used as a surrogate for education and socio-economic status. The WIMD is the Welsh Government’s official measure of relative deprivation and is calculated through eight separate domains which provide a rank of approximately 1600 postcodes across Wales according to results of the most recent national census [21].

Data collected from the survey instrument was manually transcribed into a database and pairwise deletion of missing data was performed. Data were analysed using SPSS^®^ version 27.

Continuous data was summarised using means and standard deviation, with categorical data being summarised using percentages, median (IQR) and mode where appropriate. WIMD data was categorised into deciles with 1 being the most deprived and 10 being the least. Chi-squared tests for independence was used to assess significance between categorical variables. Simple logistic regression was used for continuous variables.

Likert scales were reverse weighted where needed in order to produce homogenous scores. Scores were summarised using median and IQR. Stacked bar charts were generated using Microsoft Excel^®^ to allow for visualisation of responses.

### Qualitative analysis plan

All interviews were recorded and transcribed. Any potential identifiers noted during the transcription process, such as people or places, were removed and replaced with pseudonyms such as initials and generalised terms. Participants were coded by numbers, linked to their unique study identifier.

Inductive thematic analysis, as defined by Braun and Clarke was performed. Transcripts were read and coded according to the topic discussed [22]. Codes were grouped into themes and this was supported using NVIVO^®^ software. This process was conducted independently by one researcher with 6 of the transcripts double coded by a second independent researcher. Coding structures were then discussed with a final coding structure agreed upon. Identified themes were then further defined and named.

## Results

The survey was returned by 120 of the 332 potential participants, with a response rate of 36.1%.

Most participants were male (55% *n* = 66) and the mean age of participants was 65.98 years (SD 11.58, range 29–93). The median WIMD decile of participants was 8, (IQR 4–10, mode 10). Of the 120 participants, 30% (*n* = 36) had emergency surgery. For 75% (*n* = 90) of participants, surgery was for malignancy. Forty-five participants (37.5%) had a diagnosis of incisional hernia. The mean time from operation to completing the survey was 40.7 months (SD 18.04, range 15.3–76.5). Non-respondents were younger (mean 63.8 years), and had higher rates of emergency surgery (54% vs. 30%), but were otherwise comparable. Sample demographics can be seen in Table 1.

**Table 1:** Participants’ demographics

	Mean (SD)	Range	Non-respondents
Age	66.0 (11.58)	29–93	63.0 (15.93)
Length of time from surgery to questionnaire (years)	3.39 (1.39)	1.28–6.38	
WIMD score (Median)	7 (IQR 4–10)	2–10	8 (IQR 4–10)
	**Respondents** (*n* = 120)	**Non-respondents** (*n* = 213)	
Male Gender	66 (55%)	113 (53%)	
Operation for malignancy	90 (75%)	169 (79%)	
Emergency operation	36 (30%)	114 (54%)	
Incisional hernia diagnosis	45 (37.5%)	69 (32%)	

### Prior knowledge and recall

No prior knowledge of incisional hernia before their operation was reported by 68.3% of participants. Only 26.7% (*n* = 32) of participants could recall being told that incisional hernia was a risk following their operation. This did not correlate with gender, elective surgery, subsequent diagnosis of incisional hernia or participants with malignant disease **(**Table 2).

**Table 2:** The correlation of variables with awareness of incisional hernia as a risk of an operation

Variable	Number aware of IH as a risk of their operation (*n*)	X2 value	*P* value
Subsequent diagnosis of Incisional hernia	25	3.15	0.076
Male sex	17	1.96	0.161
Elective surgery	24	2.44	0.118
Malignant disease	25	1.91	0.167
	**b-coefficient**	**95%CI**	**P value**
Age	0.927	−0.03 to 0.02	0.202

#### Knowledge of surgical mesh

**Fig. 1:**
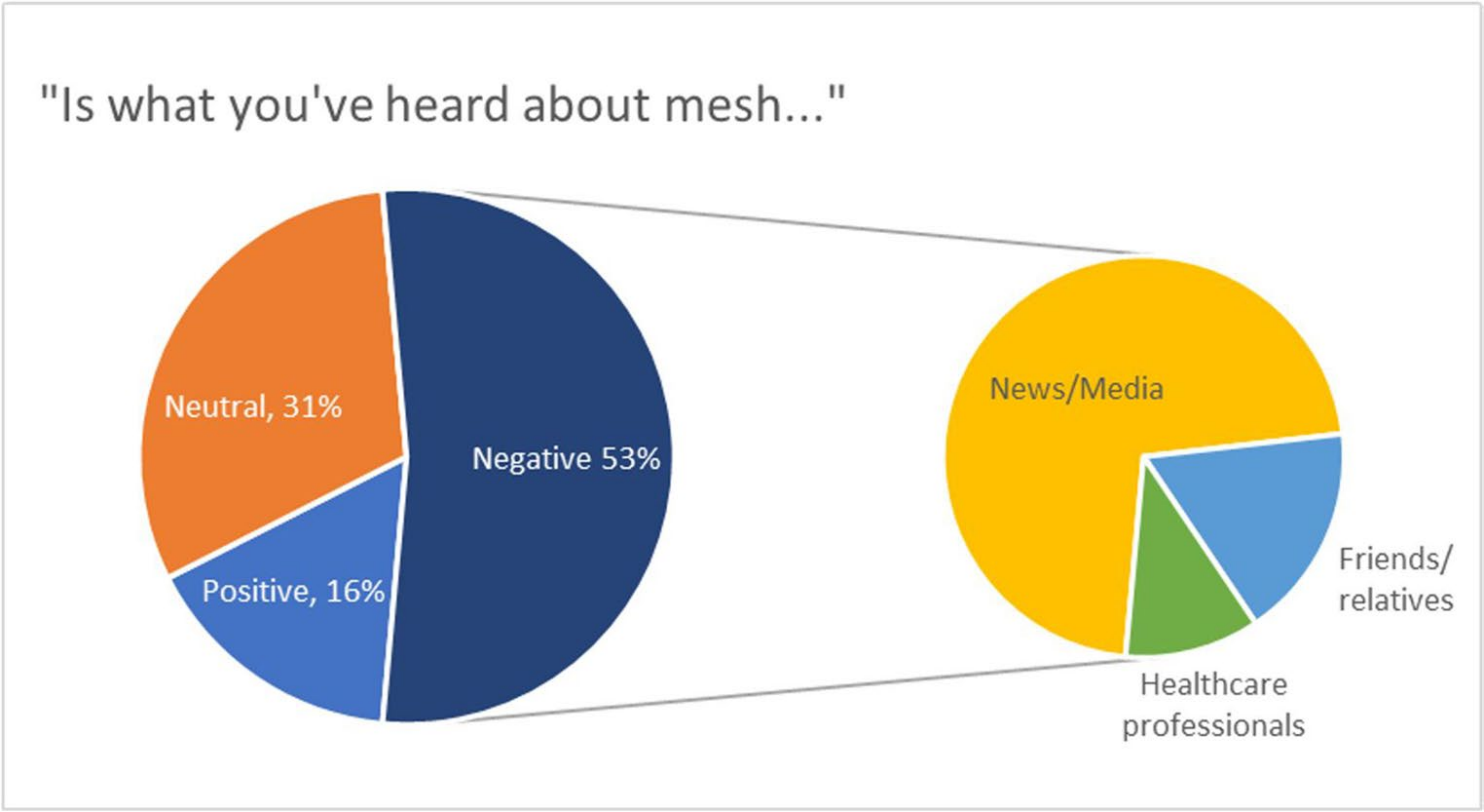
A bubble within a bubble graph of patient’s prior knowledge of mesh and the source of their information

With respect to prior knowledge of mesh, 61.3% (*n* = 73) of participants had heard of doctors using mesh. Of these, 53.9% felt that what they had heard was negative, compared to 15.7% which were positive (Fig. 1). Of the negative responses, the majority (72%) reported hearing about mesh from news/media sources.

## Concerns about mesh

**Fig. 2:**
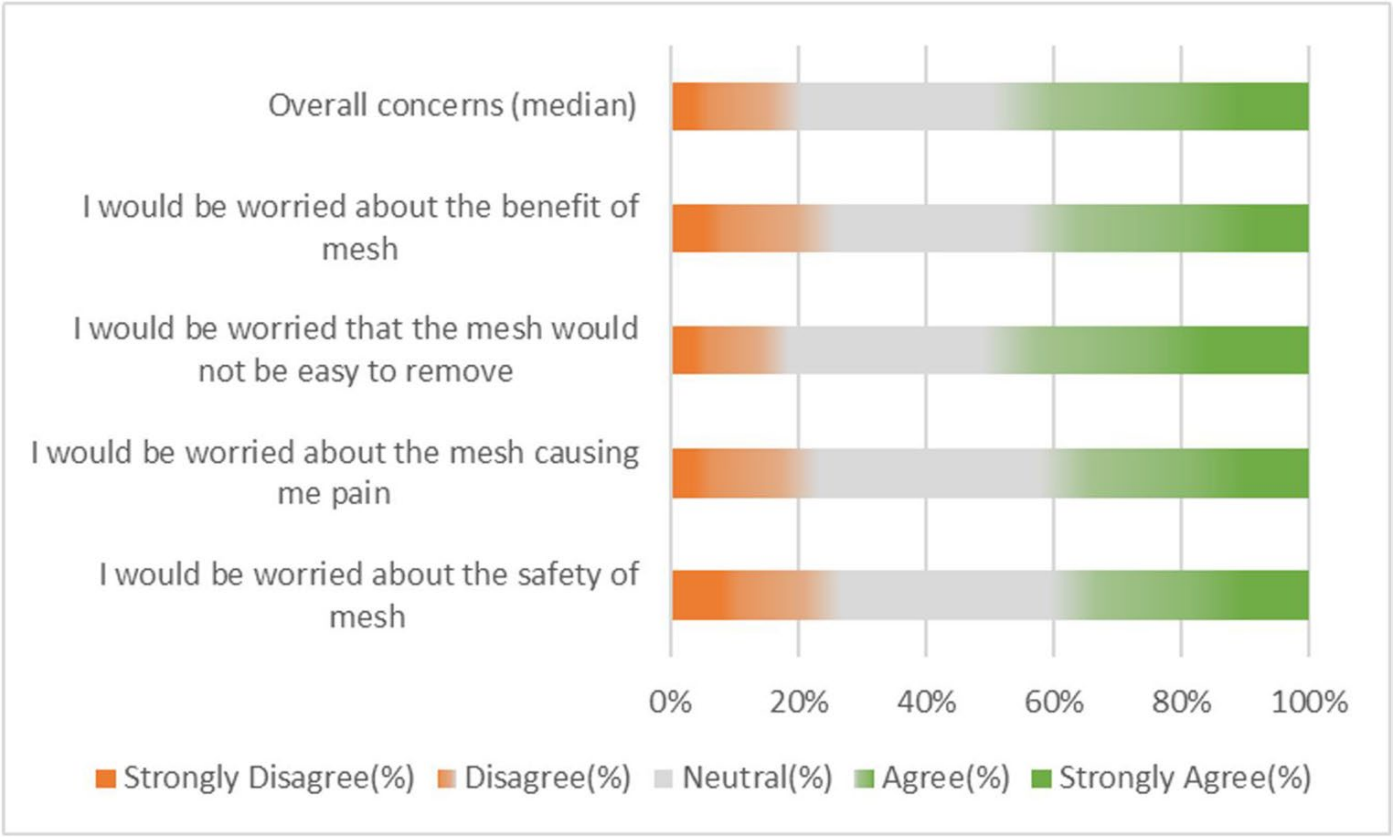
A 100% stacked bar chart showing breakdown of responses to questions about mesh

Half of participants (50%) had concerns about mesh, with 20% having no concerns at all (Fig. 2). 40% of participants were worried about the safety of mesh with similar numbers (42%) being concerned about the mesh causing them pain. Half of participants surveyed (51%) were concerned the mesh would be difficult to remove should it not work, and 45% were worried about the benefit mesh might provide.

### Risk-predictive tools and acceptability

**Fig. 3:**
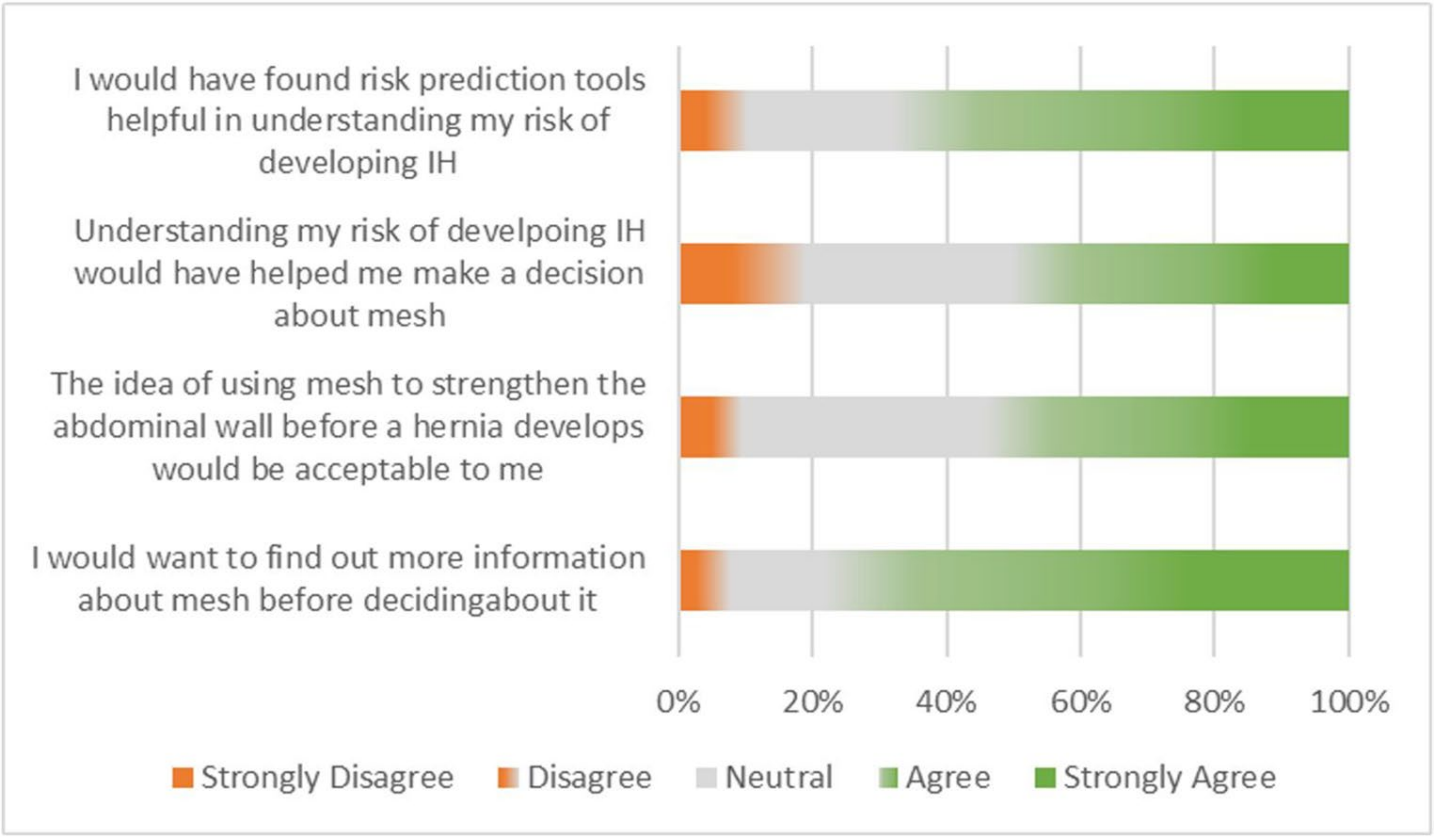
A 100% stacked bar chart showing patient responses to risk prediction and information about mesh

Most participants (69%) felt they would have found predictive tools useful in understanding their risk of incisional hernia, with 50% feeling that predictive tools would have been useful in helping them decide about prophylactic mesh. Most participants (78%) felt they would need more information about mesh before deciding about it. Despite concerns regarding mesh, only 9% of participants felt that prophylactic mesh would not be acceptable to them, with the majority (55%) feeling it would be acceptable (Fig. 3).

### Qualitative results

Twelve individuals participated in semi-structured interviews to discuss their thoughts on prophylactic mesh. Their demographics and clinical history can be seen in Table 3. Participant identifies can be seen at the end of each quote.

**Table 3:** Demographics of the interview participants

Patient ID	Age at time of response	Gender	Nature of surgery	Malignancy or benign	Post-operative Incisional hernia
01	73	Male	Elective	Malignancy	Yes
02	70	Male	Elective	Malignancy	Yes
03	79	Male	Elective	Malignancy	No
04	68	Female	Emergency	Malignancy	No
05	62	Male	Elective	Malignancy	Yes
06	58	Male	Elective	Malignancy	Yes
07	66	Male	Emergency	Malignancy	Yes
08	29	Female	Emergency	Malignancy	No
09	48	Female	Emergency	Benign	No
10	58	Female	Emergency	Benign	Yes
11	67	Male	Elective	Malignancy	No
12	73	Male	Elective	Malignancy	Yes

Following thematic analysis, three overarching themes were identified:

- Knowledge and understanding of mesh.
- Acceptability of mesh.
- Shared decision making.

**Table:** 

*“I think I've heard most about it [mesh] to do with bladder problems and it's more as I described it, almost like a sling that helps to support the pelvic muscles.” *(**10**)	

This theme focussed on interviewee’s awareness of surgical mesh and the sources of information. Its findings aligned with those of the qualitative data, demonstrating awareness of surgical mesh as a concept, predominantly in uro-gynaecological surgery.

Participants’ knowledge and understanding of “surgical mesh” were wide ranging. As seen in the quantitative data, interviewees were aware of mesh use in both hernia surgery and other types of surgery. Overall, there was an awareness of the reasons for using surgical mesh, yet it was also clear that awareness of the composition of mesh among participants was variable:

*“It’s* [mesh] *a very positive term for me*,* because the kind of thing that I imagine would strengthen the fault. (…).I think the concept is great. I think*,* the concept of putting in extra strength inside the stomach I think it’s a great idea*,* sure.”* (**02**).

*“I’m guessing that what they had inserted was some kind of metal where I guess now is probably some kind of carbon fibre.”* (**07**).

Questions surrounding perceptions of mesh supported findings in the quantitative data. Of those participants who were familiar with mesh, data indicated that personal experience notwithstanding, sources of information were primarily the media, personal contacts and even work. Across the data however, negative perceptions of mesh, particularly in relation to gynaecological surgery, were evident and predominant:

*“They use it in ladies when they do repairs down below*,* and I’ve heard some horror stories about it. In fact*,* a colleague of mine*,* his wife had some mesh fitted and they’ve had to take it out because she’s in severe pain and she’s ended up paralysed from the waist down as a result of it.”* (**06**).

*“I did listen to programs where women had mesh and ran into terrible difficulties*,* due to hysterectomies maybe*.” (**05**).

#### Acceptability of mesh

**Table:** 

*“If the consultant said, ‘we recommend that we use mesh. This will limit the possibility of help, limit the possibility of getting a hernia’ yeah, I'm going to say ‘go ahead’.”* (**11**)	

This theme centred around factors that would both positively and negatively affect the acceptability of mesh to interviewees. The desire for more information about mesh was evident throughout this theme, specifically regarding benefit and potential complications of mesh.

Despite the concerns about mesh detailed above, most participants indicted that the idea of a prophylactic mesh to prevent hernias was acceptable, in keeping with findings in the quantitative data. Acceptability was also connected with surgeons’ endorsement of mesh together with participants’ trust in their surgeons to do the best for them. As one participant put it:

*“I would have just left it to the surgeon. If he thinks it’s good for me then carry on. Do it.”* (**01**).

The acceptability of mesh was also connected with participants’ understandings of incisional hernia as a risk, and what mesh would provide in terms of risk reduction:

*“If that had been put to me*,* you’ve got a one in four chance of developing hernia but if you have mesh one in 10 chance*,* I would have probably said I want to go with the mesh.”* (**05**).

Nonetheless, reservations about mesh remained. In particular, some participants voiced concerns about the mesh related complications, including the risk of infection and the potential for pain and feared something going wrong:

*“I would feel really nervous about having a mesh because it’s a foreign thing put in my body*,* and I know about the whole vaginal mesh issue and I would be worried that something would go wrong with it.”* (**08**).

### Shared decision-making

The shared decision-making theme related to the clinician-patient relationship and how information was conveyed. This was split into three subthemes: “Context”, “Delivery” and “Content”. These reflect the participants desire to have more information regarding mesh prophylaxis, and the manner in which it could be delivered.

#### “Context”

**Table:** 

*“…..my concern is whether I would get the full operation, get the stoma, or get a reversible thing, you know….. It was how I would live without a bowel. The hernia business wasn’t any of my concern at all.”* (**02**)	

The Context subtheme focused on the co-existing factors that some participants had which limited their ability to process information.

Participants described consultations in which they had just been given a cancer diagnosis, told they needed a stoma, or were undergoing an emergency procedure. These participants felt that even if the mesh were explained to them prior to surgery, they wouldn’t have had the capacity to take in the extra information fully, and this would have negatively impacted the acceptability of mesh to them. This was not identified in the quantitative data and appears as a key factor in the acceptability of mesh to patients.

*“It was a big op you know what I mean? You get told you’ve got cancer and things tend to go in one ear and out the other. You’re in a world of your own sometimes.” (****01****)*.

***“*** *I think that because my op was emergency … and there was a lot of overwhelming stuff going on and to know the hernia risk would have just been an extra thing for me to process.” (* ***08*** *)*

#### “Delivery”

**Table:** 

*“I’d like perhaps to have some case studies from people like me that have a hernia and are living with a hernia……Or YouTube® videos or something”*. (**06**)	

This subtheme focused on the delivery of information about mesh, in particular, the medium in which information should be provided to patients.

Participants discussed use of a physical form of the information such as patient information leaflets or through previous participants’ experiences. This subtheme echoed findings in the quantitative data that supported patient’s desire for more information on mesh and IH, alongside a desire to be involved in the decision-making process.

“*Possibly a small pamphlet along with the chat with the Surgeon. I would think that would be for me the most helpful way of doing it*”. (**03**)

#### “Content”

**Table:** 

*“I don't feel I know enough about it. And fine. It could be OK for the first year. Two years, five years. But what happens 10 years down the line or 15 or 20?” *(**10**)	

The “Content” subtheme focussed on the nature of information that participants would like to receive, and is linked to factors identified in the “Acceptability of mesh “ theme described above.

This subtheme identified information that participants would like to receive about mesh that would influence their decision making around this, such as likelihood of success and potential complications.

***“****If I’d have the time and the opportunity. I’d have probably said*,* what are the drawbacks? Does anything go wrong with it?” (****07****)*.

## Discussion

This study sheds light on patient’s views of mesh prophylaxis and identifies factors that influence acceptability of mesh. These factors can be targeted at a local, national or even international level to aid in the implementation of mesh-augmented abdominal wall closure and reduce incisional hernia rates. The results of this study demonstrate little pre-operative awareness of incisional hernia as a risk of surgery amongst participants. There was a predominance of negative preconceptions towards surgical mesh, often driven by the media yet in spite of this, mesh prophylaxis was acceptable to participants in this study. Factors that affect acceptability involved including patients in the decision-making process and relaying information to them in a manner that they could understand and process.

In this study, participants who had undergone abdominal surgery were asked about their knowledge of incisional hernia and surgical mesh. When asked about the consent process, two thirds had no knowledge of incisional hernia prior to their operation. Given that the mean time from operation to completing the questionnaire was 3 years, this is perhaps not surprising. Yet our results showed no correlation between being diagnosed with incisional hernia and positive recall, nor in age, sex or nature of operation or if the patient had benign or malignant disease, suggesting that recall was similar between all participants. Considering incisional hernia is the most frequently occurring complication of abdominal surgery, it is concerning that this may not be explained to patients in a manner that they can recall.

The results of this study indicated that participants’ did have preconceived ideas about mesh, with 53% indicating a negative attitude towards mesh as a term in general. The majority of these negative views were being driven by media sources, indicating that negative media coverage is impacting our patient population. The identification of negative perceptions of mesh as a theme in the qualitative data enhances this finding, with participants in the qualitative phase of the study frequently referencing negative media sources. Given the extent and reach of social media, this influence is to be expected. In an analysis of posts regarding hernia mesh on two social media platforms, Fadaee et al. demonstrated that 39% of posts about hernia mesh on the social media platform “X^®^” were negative, whilst on Facebook^®^ this rose to 95% [14]. In spite of these preconceptions, it appears that participants were able to differentiate between mesh use in uro-gynaecological procedures and mesh in hernia surgery, with the majority of negative comments relating to the use of mesh in prolapse surgery.

Our results showed that most participants had concerns about all domains of mesh, with only around 20% having no concerns at all. Despite these concerns however, mesh prophylaxis was not acceptable to only 9% of participants, and qualitative data supported the overall acceptability of mesh prophylaxis to participants. Our results suggested that despite negative preconceptions towards mesh, if given time to discuss and the right information, patients would be willing to consider mesh prophylaxis.

Explaining risk of developing incisional hernia to patients certainly appeared to be a factor in determining acceptability of mesh, with 69% of our survey participants indicating they would have found a risk predictor useful in understanding their risk of developing IH; a finding supported by the qualitative data. Mesh prophylaxis is not acceptable to, nor suitable, for all patients and current EHS guidelines suggest that prophylaxis should be targeted to “High risk” patients [10]. How surgeons define “High risk” and how we communicate this to our patients will certainly impact the uptake of mesh prophylaxis, and future studies should be conducted with surgeons and patients to identify differences in risk profiles between the two groups.

Whilst the quantitative and qualitative data suggested a desire for more information about mesh, it was surprising to see the “context” subtheme emerge from the qualitative data. This suggests that a pre-operative discussion about mesh would have been too much information to process for patients, particularly at the time of cancer diagnosis or in an emergency setting.

Research into how patients process information has demonstrated that 40–80% of what a clinician explains is forgotten immediately after the consultation, and that this percentage increases in conjunction with the volume of information [23, 24]. This can be reduced even more when associated with “attentional narrowing”, whereby patients fixate on emotional or physical distress further limits additional capacity to process information; seen in our study as those that reported only remembering being told they had cancer or that they might need a stoma [25]. Moving forward, attention needs to focus on how we present information on risk-benefit to patients and allowing them the time and resource to think about it. Further work should focus on development of patient-centred information, not only for mesh-prophylaxis but also for other peri-operative topics such as prehabilitation, stoma care and enhanced recovery programmes. The results of this study have the potential to influence information delivery across medical specialties and should not be seen as simply relating to incisional hernia prevention.

There are limitations within this study that need acknowledgment. A limitation of mixed-methods studies is response bias and generalisability of results to the wider population. Response rates for postal surveys vary in the literature from 20 to 70% [26], and our response rate of 36.1% whilst acceptable, leaves potential for bias. Potential participants with strongly negative views towards mesh may have decided to ignore requests to participate and this may affect the generalisability of findings.

Level of participant education may also affect the generalisability of results. The national measure of deprivation used was low in our group, with a median decile of 8 and a mode of 10, implying a higher-than-average level of education in our cohort.

The population choice population of patients who have undergone surgery, rather than at random from the population may also affect the generalisability of the findings, however we considered this group of participants best-placed to explore the mindset our patients have at the time of surgery, which we feel encouraged deeper, more meaningful conclusions that have potential to impact patient care.

## Conclusion

Mesh prophylaxis is broadly acceptable to patients despite pre-existing concerns regarding mesh, which are driven by negative media coverage. Factors that influence acceptability are involvement of the patient in the decision-making process, and the way information is delivered to patients. It is important for clinicians to recognise the scenarios that might present information overload at the time of decision making for surgery in patients and to counter this by developing patient-centred resources to aid in information delivery.

## Supplementary Information

Below is the link to the electronic supplementary material.

Supplementary Material 1 (DOCX 73.4 KB)

Supplementary Material 2 (DOCX 34.4 KB)

Supplementary Material 3 (DOCX 30.7 KB)

## Declarations

L Smith has received paid consultancy work from BD Uk unrelated to this project.

### Trial registration

IRAS: 310695, ClinicalTrials.gov: NCT05384600
