# Supplementary material for: The invite study: incisional hernia prevention: prophylactic mesh from the patient’s perspective
Source: Hernia. 2025 Sep 4;29(1):272. doi: 10.1007/s10029-025-03463-z (PMC12411575; doi:10.1007/s10029-025-03463-z)
Supplement: Supplementary file 1 — Supplementary Material 1 (DOCX 73.4 KB) [file 10029_2025_3463_MOESM1_ESM.docx]

INVITE

Incisional Hernia Prevention: Risk-benefit from a patient’s perspective

Participant Questionnaire

Version 1.0, 11/05/2022

**Please circle your choice of answer in each case.**

**Section 1: Background**

We are trying to understand a bit more about you and the effects of your operation on your health.

What is your height? _______ cm / ft What is your weight? _______ Kg / Stone

Have you ever smoked on a daily basis?

Yes, currently a smoker Yes, but an ex-smoker Never Smoked

Do you currently feel pain at the site of the scar from your operation?

Yes No Sometimes

Do you ***feel*** a swelling or bulge at the site of your scar?

Yes No Sometimes

Do you ***see*** a swelling or bulge at the site of your scar?

Yes No Not sure

**Section 2: Knowledge of Incisional Hernia**

After having abdominal surgery, there is a risk that some of the abdominal contents can push through a weakness left in the muscle at the site of the operation. This is called an incisional hernia.

Did you know what an incisional hernia was before your first operation?

Yes No Don’t know/Unsure

Were you told that Incisional Hernia was a risk for your operation?

Yes No Unsure/Don’t know

How much information regarding incisional hernia was given to you before the operation?

None Not enough The right amount Too much

Have you heard of doctors using mesh as part of a hernia repair?

Yes No Don’t know/Unsure

Is what you’ve heard about mesh...

Positive Negative Neutral Other (please record comments below)

Do you know someone who has had a hernia repair?

Yes No

If yes, did it involve mesh?

Yes No Don’t know/unsure

Was their outcome positive or negative?

Positive Negative Not sure

If you have heard of mesh, where have you heard about it from?

Doctor/Healthcare professional News/Media Friend/relative

Other:__________ Not applicable

| If you have any other comments about mesh, please feel free to record them below. |
| --- |

**Section 3: Risk and prevention**

What is a risk-prediction tool?

Risk-prediction tools are used by doctors to work out a person's risk of developing a medical condition, for example the risk of having a heart attack based on the risk factors that they have. This allows doctors to convey the risk to patients in the form of a number, for example 10% or 1-in-10.

Risk-prediction and Incisional Hernia

Risk-prediction tools are being developed with the aim of working out a person’s risk of developing an incisional hernia **before** their operation. We hope that this will allow surgeons to give patients an idea of what their risk is before the operation. Patients can then understand if they are at high, medium or low risk, and what they might be able to do about it before the operation.

For patients that are predicted to be “high risk” for developing an incisional hernia, it may be possible to use a synthetic mesh, similar to those used to fix groin hernias. This would be placed in the wound at the end of the initial operation to strengthen the wound to try and reduce the chance of developing an incisional hernia.

Aims of the study

We want to know whether mesh placed to prevent hernias during the initial surgery would be acceptable to patients, and if patients would find a risk-prediction tool helpful when learning more about risk of incisional hernia before surgery.

Please read the questions below and circle the answer that best applies to you.

Q1. If you were told before your operation that you were “high risk” of developing an incisional hernia, and that using mesh might help to reduce that risk, to what extent would you agree or disagree with the following statements?

1. **“I would be worried about the safety of mesh”**

| 1 | | 2 | | 3 | | 4 | | 5 | |
| --- | --- | --- | --- | --- | --- | --- | --- | --- | --- |
|  |  |  |  |  |  |  |  |  |  |
| Strongly disagree | | Disagree | | Neutral | | Agree | | Strongly agree | |

1. “**I would be worried about the mesh causing me pain”**

| 1 | | 2 | | 3 | | 4 | | 5 | |
| --- | --- | --- | --- | --- | --- | --- | --- | --- | --- |
|  |  |  |  |  |  |  |  |  |  |
| Strongly disagree | | Disagree | | Neutral | | Agree | | Strongly agree | |

1. **“I would be worried that if the mesh was implanted, it would not be easy to remove at a later date if it didn’t work”**

| 1 | | 2 | | 3 | | 4 | | 5 | |
| --- | --- | --- | --- | --- | --- | --- | --- | --- | --- |
|  |  |  |  |  |  |  |  |  |  |
| Strongly disagree | | Disagree | | Neutral | | Agree | | Strongly agree | |

1. **“I would be worried about how much benefit I will get from mesh”**

| 1 | | 2 | | 3 | | 4 | | 5 | |
| --- | --- | --- | --- | --- | --- | --- | --- | --- | --- |
|  |  |  |  |  |  |  |  |  |  |
| Strongly disagree | | Disagree | | Neutral | | Agree | | Strongly agree | |

1. **“I do not think I have enough information about mesh to make a decision about it”**

| 1 | | 2 | | 3 | | 4 | | 5 | |
| --- | --- | --- | --- | --- | --- | --- | --- | --- | --- |
|  |  |  |  |  |  |  |  |  |  |
| Strongly disagree | | Disagree | | Neutral | | Agree | | Strongly agree | |

Q2. Thinking back to your original operation, please read the questions below and circle the answer that best applies to you.

1. **“I would have found risk-scoring before an operation useful in helping me understand my risk of developing incisional hernia”**

| 1 | | 2 | | 3 | | 4 | | 5 | |
| --- | --- | --- | --- | --- | --- | --- | --- | --- | --- |
|  |  |  |  |  |  |  |  |  |  |
| Strongly disagree | | Disagree | | Neutral | | Agree | | Strongly agree | |

1. **“Understanding my risk of developing incisional hernia would have helped me to make decisions about different treatment options”**

| 1 | | 2 | | 3 | | 4 | | 5 | |
| --- | --- | --- | --- | --- | --- | --- | --- | --- | --- |
|  |  |  |  |  |  |  |  |  |  |
| Strongly disagree | | Disagree | | Neutral | | Agree | | Strongly agree | |

1. **“The idea of using mesh to strengthen the wound before a hernia develops would be acceptable to me”**

| 1 | | 2 | | 3 | | 4 | | 5 | |
| --- | --- | --- | --- | --- | --- | --- | --- | --- | --- |
|  |  |  |  |  |  |  |  |  |  |
| Strongly disagree | | Disagree | | Neutral | | Agree | | Strongly agree | |

1. **“I would want to find out more information regarding mesh before deciding if it would be acceptable to me”**

| 1 | | 2 | | 3 | | 4 | | 5 | |
| --- | --- | --- | --- | --- | --- | --- | --- | --- | --- |
|  |  |  |  |  |  |  |  |  |  |
| Strongly disagree | | Disagree | | Neutral | | Agree | | Strongly agree | |

| **What additional information about mesh would you want to know in order to make a decision about it?**  *Please record your answer in the box below* |
| --- |

**Thank you for taking the time to complete this questionnaire.**

If you have any further comments about any of the topics discussed, please feel free to contact the research team phone on 02921 842934 or email ColorectalResearch.CAV@wales.nhs.uk.
