# Supplementary material for: The invite study: incisional hernia prevention: prophylactic mesh from the patient’s perspective
Source: Hernia. 2025 Sep 4;29(1):272. doi: 10.1007/s10029-025-03463-z (PMC12411575; doi:10.1007/s10029-025-03463-z)
Supplement: Supplementary file 3 — Supplementary Material 3 (DOCX 30.7 KB) [file 10029_2025_3463_MOESM3_ESM.docx]

INVITE Interview questions

| INVITE ID: _ |
| --- |
|  |
| Interview details |
| Date/ time: |
| Location: |
| Interviewer: |
|  |
| Use these questions to guide the qualitative interview.  Ensure that the recording device is switched on and has sufficient battery to last the duration of the interview.  The participant may be offered breaks or end the interview at any time. |

*We have invited you for a chat today to discuss the use of mesh to prevent a hernia (or bulge in the tummy wall) that can happen after people have a cut in their tummy wall as part of their operation. There has been some work done and evidence is starting to build that mesh could be placed on the tummy wall at the end of a planned operation to reduce the chance of a hernia developing. We want to know how patients feel about that and specifically how you feel about that today.*

- What do you understand by the term surgical mesh
- What do you think about the term mesh/surgical mesh
- What have you heard/how much do you know about mesh in relation to hernias.
- What do you think the risk of developing a hernia after surgery is on average?

(*0-100 scale / %)*

- How would you feel if you were offered mesh at the time of your operation?
- What would you like to know about mesh before deciding if you wanted to have it?
- Would you think more about the risks of mesh, or the benefit that you might gain from it?

“Surgeons are starting to be able to predict who is at risk of developing hernias after surgery.”

- At what level of risk for developing a hernia would you be prepared to consider mesh? <1%, 5%, 10%, 25%, >50%, Would not accept it (1 in 100, 1 in 50, 1 in 25, 1 in 10, 1 in 5, 1 in 2)

“The risk of mesh being used in this way is still being investigated. Potential risk include infection, pain and the need for removal of mesh”

- At what risk of complications would you not consider mesh? <1%, 5%, 10%, 25%, >50%

(*Or 0-100 scale?)*

*(1 in 100, 1 in 50, 1 in 25, 1 in 10, 1 in 5, 1 in 2)*
